# Development of tri-cistronic CLDN18.2 CAR-T cells incorporating PD-1/CD28 switch and cyclophilin A for enhanced solid tumor immunotherapy

**DOI:** 10.71150/jm.2510017

**Published:** 2026-01-31

**Authors:** Heon Ju Lee, Seo Jin Hwang, Eun Hee Jeong, Mi Hee Chang, Bu Yeon Heo, Jaeyul Kwon, Yoona Noh, Jihoon Nah

**Affiliations:** 1 CARBio Therapeutics Co., Ltd., Cheongju 28160, Republic of Korea; 2 Translational Immunology Institute, Department of Medical Education, College of Medicine, Chungnam National University, Daejeon 35015, Republic of Korea; 3 Department of Biological Sciences and Biotechnology, Chungbuk National University, Cheongju 28644, Republic of Korea; 4 Department of Biochemistry, Chungbuk National University, Cheongju 28644, Republic of Korea

**Keywords:** tri-cistronic CAR-T cells, Claudin18.2 (CLDN18.2) antigen, PD-1/CD28 chimeric switch receptor, cyclophilin A (CypA), gastric cancer, immune checkpoint modulation

## Abstract

Chimeric antigen receptor (CAR)-T cell therapy holds significant potential for the treatment of solid tumors. However, immune suppression and tumor-specific barriers limit its application. Claudin 18.2 (CLDN18.2), a gastric lineage-specific tight junction protein highly expressed in gastric and pancreatic cancers, is a promising therapeutic target. In this study, we aimed to develop a next-generation tri-cistronic CLDN18.2-directed CAR-T cell platform that integrates a programmed cell death protein 1 (PD-1)/CD28 chimeric switch receptor with cyclophilin A (CypA). This platform sought to counteract PD-1–mediated immunosuppression and enhance T-cell activation and persistence. We generated CLDN18.2 CAR-T cells incorporating costimulatory inducible T-cell costimulator (ICOS) domains using lentiviral vector-based recombinant engineering. We further evaluated their cytokine release, cytotoxic activity, and safety profiles. In vitro, tri-cistronic CAR-T cells exhibited markedly increased interferon γ and tumor necrosis factor α secretion and enhanced cytotoxicity against CLDN18.2-positive gastric cancer cells compared with conventional CAR-T constructs. In vivo, these cells showed superior antitumor efficacy and sustained tumor regression without observable toxicity in xenograft gastric cancer models. Collectively, these findings demonstrate that the integration of PD-1/CD28 signaling and CypA within a tri-cistronic framework significantly reinforces CAR-T cell functionality and durability. This suggests strong clinical potential as a next-generation immunotherapy for solid tumors.

## Introduction

Recent advances in chimeric antigen receptor (CAR)-T cell therapies have improved outcomes for patients with previously intractable diseases. In particular, considerable remission rates have been observed in patients with hematologic malignancies. The application of these therapies has also expanded to include autoimmune diseases and solid tumors (Baker and June, 2024; Jaspers et al., 2023; June and Sadelain, 2018). CAR-T technology, which delivers therapeutic genes into T cells via viral vectors, has rapidly progressed from clinical trials to multiple approved therapies worldwide. Thus, it is an excellent candidate for next-generation gene and immune cell therapies (Maus and June, 2016).

Immune homeostasis is maintained by a tightly regulated balance between effector function and self-tolerance. Its centrality has been markedly underscored by recent breakthroughs in regulatory T-cell (Treg) biology. The 2025 Nobel Prize in Physiology or Medicine, which honored the discovery of Tregs and identification of FOXP3 as their lineage-defining transcription factor, signified a formal recognition of this paradigm shift (Brunkow et al., 2001; Ramsdell, 2003; Sakaguchi et al., 2006). Tregs enforce peripheral immune tolerance, prevent autoimmunity, and modulate inflammatory responses. Genetic or functional deficits in FOXP3 lead to severe immune dysregulation and multi-system disorders. Thus, mechanistic insights into FOXP3-driven networks and their integration with environmental and signaling cues have established a foundation for next-generation immunotherapeutic strategies. This expanding knowledge base directly informs the design of engineered cell therapies, including multifunctional CAR-T platforms that necessitate the precise control of immune regulation within the tumor microenvironment.

**Fig. 1.**
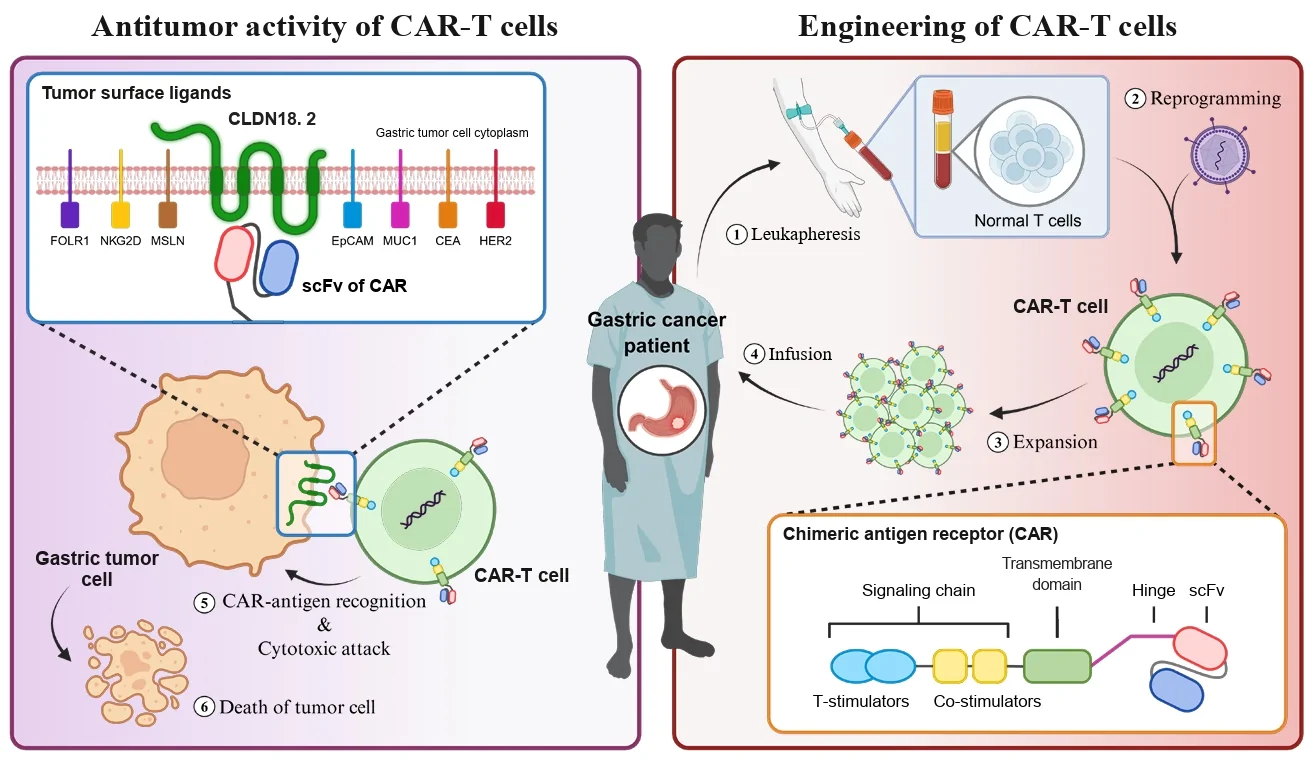
Engineering and antitumor activity of CAR-T cells targeting gastric tumor antigens. Autologous T cells are collected from a patient with gastric cancer via leukapheresis (①) and genetically modified (②) to express a chimeric antigen receptor (CAR) comprising scFv, hinge, transmembrane, and intracellular signaling domains. The engineered CAR-T cells are subsequently expanded ex vivo (③) and re-infused into the patient (④). Upon encountering gastric tumor cells, CARs specifically bind to tumor-associated ligands such as CLDN18.2 on the cell surface (⑤), consequently triggering CAR-T–cell activation and the cytotoxic elimination of tumor cells (⑥) through perforin/granzyme-mediated apoptosis. Created in BioRender. jo, y. (2025) https://BioRender.com/4nvod65.

Of the advanced gene and cell therapies developed to date, CAR-T cell therapy is a prime example of engineered immunotherapy (Fig. 1). Both ex vivo and emerging in vivo approaches use viral vectors to reprogram mature T cells for clinical use. The development of next-generation CAR-T cells that target refractory hematologic malignancies, solid tumors, and autoimmune diseases has facilitated the design of multifunctional constructs capable of overcoming the immunosuppressive tumor microenvironment (Patel et al., 2020; Tahir, 2018). Since 2015, strategies such as bispecific CARs, transcriptional regulatory element design, tyrosine kinase inhibitors, and oncolytic virus combinations have rapidly evolved to enhance therapeutic efficacy against solid tumors (Ghartey-Kwansah et al., 2018; Lynn et al., 2019; Mestermann et al., 2019; Miao et al., 2021).

In this study, we replaced conventional co-stimulatory domains, such as CD28 or 4–1BB, with inducible T-cell costimulator (ICOS; CD278), which shows promising applicability in solid tumors because it can induce gradual and controlled attenuation of immune overactivation. To further enhance T-cell function and counteract immune checkpoint-mediated dysfunction, we incorporated the programmed cell death protein 1 (PD-1)/CD28 chimeric switch receptor (CSR)—expressed separately via an internal ribosome entry site—to restore T-cell activation in immunosuppressive tumor microenvironments (Li and Wang, 2020; Mazinani and Rahbarizadeh, 2022). This molecular switch was designed to simultaneously promote immune activation and improve safety by preventing T-cell suppression mediated by PD-1/Programmed Death-Ligand 1 (PD-L1) interactions. Moreover, CD28 signaling promotes T-cell proliferation and differentiation. This may enable more effective clearance of solid tumors, such as pancreatic, liver, and gastric cancers, than that of traditional CAR-T cell therapies, thereby improving remission rates (Guo and Cui, 2020; Lee et al., 2024).

## Materials and Methods

### Generation of CLDN18.2 CAR-T cells

CAR-T cells were generated as described by Lee et al. (2024). Briefly, human peripheral blood mononuclear cells (PBMCs; Zen-Bio) were thawed and incubated with CTS Dynabeads CD3/CD28 (Gibco) at a 2:1 ratio to activate the T cells. Two days after-activation, the T cells were transduced with lentiviral vectors encoding the CAR construct at a multiplicity of infection of 5 in the presence of 8 µg/ml polybrene. After 24 h, the virus was removed, and the cells were cultivated in CTS^TM^ OpTmizer^TM^ T Cell Expansion SFM (Gibco) supplemented with 5% human serum (Sigma) and 200 IU/ml interleukin 2 at 37°C in a 5% CO_2_ atmosphere for 11 days. CAR and PD-1 expression levels were determined on days 4, 6, 8, and 11 post-transduction.

### Flow cytometric analysis

CAR expression was assessed by surface staining with goat anti-mouse IgG F(ab’)_2_ (Jackson ImmunoResearch). The expression levels of CAR in CAR-T cells were normalized against Mock T cells. Claudin18.2 expression in cancer cell lines was evaluated using a recombinant human anti-Claudin 18.2 monoclonal antibody (Invitrogen), followed by detection with a goat anti-human IgG Fc secondary antibody conjugated to phycoerythrin (PE; Invitrogen). The following fluorochrome-conjugated antibodies were used to immunophenotype T cells and PBMCs: CD3-PerCP Cy5.5 (BioLegend), CD4-PE Cy7 (BioLegend), CD8-APC (BD), CD14-FITC (BD), CD45-BV421 (BD), CD56-PE (BD), CD19-APC (BD), CD25-FITC (BioLegend), and CD69-APC Cy7 (BioLegend). Data were analyzed using the Kaluza analysis software (Beckman Coulter).

### In vitro cytotoxicity assay

The cytolytic activity of CAR‑T cells was evaluated using a lactate dehydrogenase (LDH) release assay. CAR‑T cells or Mock‑T cells were co-cultured with AGS, AGS-C18.2, KATO-III, and KATO-III-C18.2 gastric cancer cells (2 × 10^4^ per well) at effector-to-target (E:T) ratios of 0.5:1 and 1:1 for 24 h at 37°C in a 5% CO_2_ incubator. Mock‑T cells were non‑transduced, bead‑activated T cells that were expanded under the same culture conditions but without exposure to lentiviral particles, allowing us to distinguish CLDN18.2‑independent background activity from CAR‑dependent cytotoxicity. After co-culture, cell-free supernatants were collected for LDH quantification using the CytoTox 96^®^ Non-Radioactive Cytotoxicity Assay kit (Promega) according to the manufacturer’s instructions. Absorbance was measured at 490 nm, and percent cytotoxicity was calculated using the following formula: Cytotoxicity (%) = (Experimental LDH – Effector Spontaneous – Target Spontaneous)/(Target Maximum – Target Spontaneous) × 100.

Each condition was assayed in triplicate, and background signals from spontaneous LDH release were subtracted to ensure accurate measurement of CAR‑T-mediated cytotoxicity.

### Cytokine release assay

To evaluate cytokine secretion, CAR‑T cells or Mock‑T cells were co-cultivated with AGS, AGS-C18.2, KATO-III, and KATO-III-C18.2 cell lines (2 × 10^4^ target cells per well) at an E:T ratio of 1:3 for 24 h at 37°C in a 5% CO_2_ incubator. Mock‑T cells were non‑transduced, bead‑activated T cells that were expanded under the same culture conditions but without exposure to lentiviral particles, allowing us to distinguish CLDN18.2‑independent background activity from CAR‑dependent cytokine release. After incubation, cell-free supernatants were collected to measure the concentration of interferon-gamma (IFN-γ) and tumor necrosis factor-alpha (TNF-α) using the Human IFN-γ and TNF-α ELISA kits (R&D systems) according to manufacturer’s instructions.

### In vivo evaluation of a xenograft model of gastric cancer

To assess antitumor efficacy in vivo, subcutaneous gastric cancer xenograft models were established using seven-week-old female NOD/Shi-scid, IL-2Rγ KOJic/NOG mice (Saeronbio, Korea). The mice were subcutaneously inoculated with 5 × 10^6^ AGS-C18.2 cells in the right flank using a BD ultra-Fine insulin syringe. After xenografts reached the models (tumor size: approximately 80 ± 10 mm^3^), Mock-T (non-transduced T cells) and CLDN18.2 CAR-T cells (CT-030, CT-001, CT-017, and CT-022 CAR-T) were intravenously inoculated into mice via the tail vein. Tumor size and body weight were measured twice per week.

### Statistical analysis

Data are presented as the Mean ± SD. Statistical significance was determined using post-hoc tests after one-way ANOVA. Statistical analyses were performed using the GraphPad Prism 10 software. Statistical significance was defined as *, **, and *** for p-values < 0.05, < 0.01, and < 0.001, respectively.

## Results

### Design and generation of CLDN18.2 CAR-T cells

**Fig. 2.**
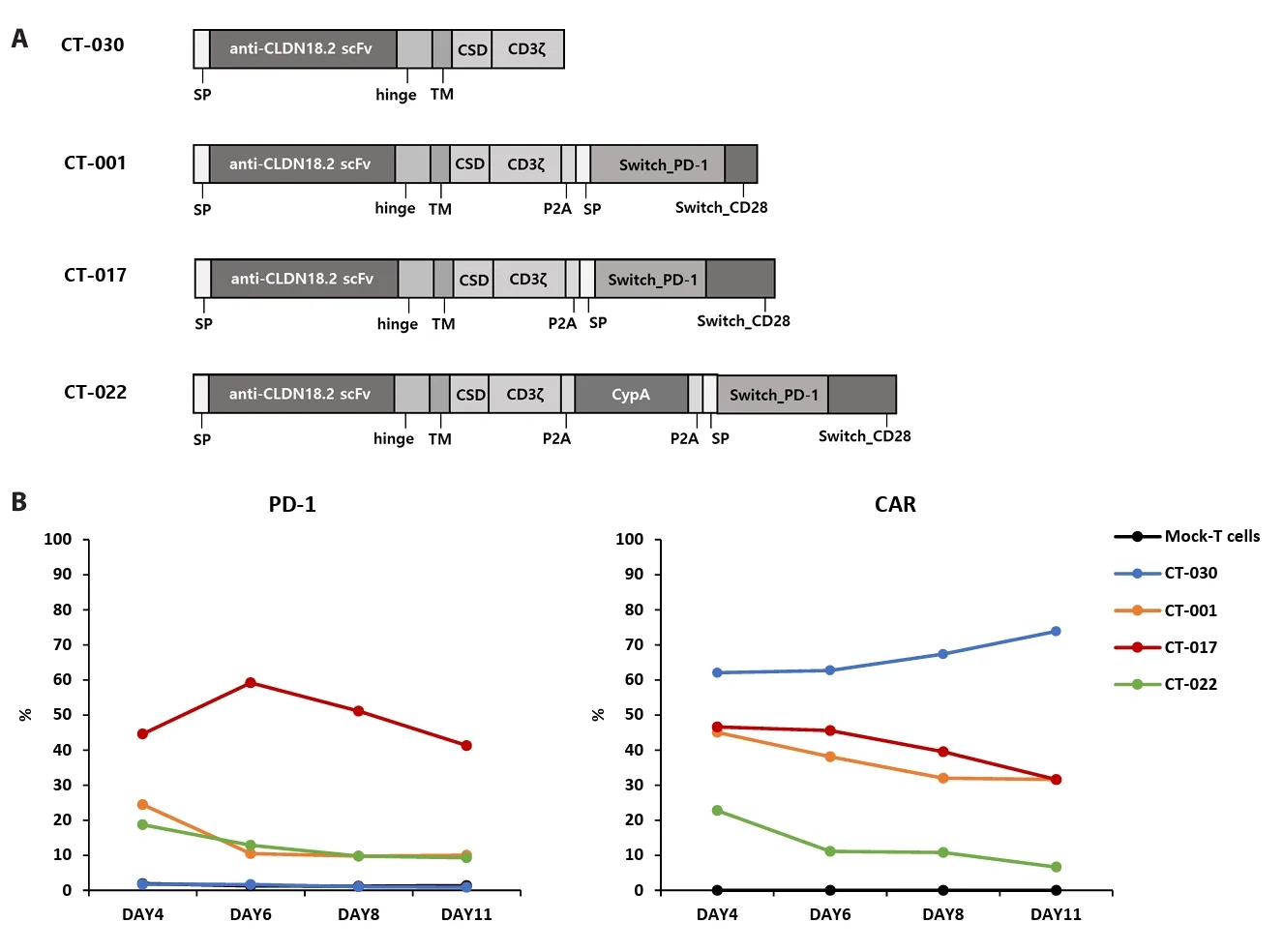
CLDN18.2 CAR constructs and the characteristics of CLDN18.2 CAR-T cells. (A) The schematic of the CAR constructs. Four individual CLDN18.2 CAR constructs were designed and engineered. CT-030 includes antigen-binding (anti-CLDN18.2 scFv), hinge (CD8α), transmembrane (ICOS), costimulatory (ICOS CSD), and stimulatory (CD3ζ) domains. The PD-1/CD28 CSR was introduced into the CT-030 construct to generate CT-001 and CT-017. CT-001 and CT-017 had the same structure, except for variations in the composition and arrangement of their PD1/CD28 CSRs. The PD-1/CD28 CSR of CT-001 was composed of an extracellular domain, a transmembrane domain derived from PD-1, and a CD28-derived intracellular domain. In contrast, the CSR of CT-017 consists of an extracellular domain derived from both PD-1 and CD28, followed by transmembrane and intracellular domains derived from CD28. CypA was further incorporated into the CT-017 backbone to generate CT-022, a tri-cistronic CAR-T construct that contained both the PD-1/CD28 CSR and CypA. SP, signal peptide; TM, transmembrane domain; CSD, costimulatory domain; SD, stimulatory domain; P2A, 2A peptide; CSR, chimeric switch receptor; CypA, Cyclophilin A. (B) Characteristics of the CLDN18.2 CAR-T. To generate CAR-T cells, activated T cells were transduced with lentiviruses carrying the CAR genes and further cultivated for 11 days. The expression levels of CAR and PD-1 in CAR-T cells were assessed using flow cytometry. CAR expression levels were adjusted using Mock-T cells as a baseline.

A series of CLDN18.2-specific CAR constructs was engineered for functional evaluation. The construct (CT-030) consists of an anti-CLDN18.2 single-chain variable fragment (scFv) as an extracellular antigen-binding domain, a CD8α hinge, an ICOS transmembrane domain, an ICOS costimulatory domain, and a CD3ζ stimulatory domain. To enhance CAR-T cell functionality, PD-1/CD28 CSRs were incorporated into CT-030 to yield CT-001 and CT-017, which possess distinct configurations of PD-1 and CD 28 gene elements. Furthermore, CT-022 was generated by introducing cyclophilin A (CypA) into the CT-017 backbone, thereby producing a tri-cistronic CAR-T construct containing both the PD-1/CD28 CSR and CypA (Fig. 2A). In this study, CypA was incorporated only into the CT‑017 backbone to generate the tri‑cistronic CT‑022 construct, which co‑expresses PD‑1/CD28 CSR and CypA. CLDN18.2 CAR‑T cells expressing CypA in the absence of PD‑1/CD28 were not generated, because our design focused on evaluating stepwise enhancement from the base CAR (CT‑030) to PD‑1/CD28 CSR alone (CT‑001 and CT‑017) and finally to the combined PD‑1/CD28 plus CypA module (CT‑022).

The synthesized DNA construct was subcloned into a third-generation lentiviral vector derived from the pLenti-EF1a backbone and designated as pCT-030, pCT-001, pCT-017, and pCT-022. CAR-T cells targeting CLDN18.2 were generated through lentiviral transduction of activated human T cells with each vector. CAR expression was monitored throughout the cell generation process; anti-CLDN18.2 CAR-transduced cells consistently demonstrated robust CAR surface expression, while minimal expression was detected in untransduced (Mock-T) controls.

PD-1 expression was also characterized in both CAR-T and Mock-T cells. Consistent with the construct design, CAR-T cells transduced with vectors encoding the PD-1/CD28 CSR exhibited markedly elevated PD-1 expression, whereas CT-030 and Mock-T cells showed minimal to no PD-1 expression on the surface (Fig. 2B). We also assessed PD-L1 expression on the gastric cancer cell lines by flow cytometry. Under basal culture conditions, PD-L1 was detected on approximately 45% of AGS cells, 10% of AGS-CLDN18.2 cells, 45% of KATO III cells, and 43% of KATO III-CLDN18.2 cells, indicating that these targets provide endogenous ligand for PD-1/CD28 signaling, although at relatively modest levels (Fig. S1).

### Cytotoxicity and cytokine release of CLDN18.2 CAR-T cells

**Fig. 3.**
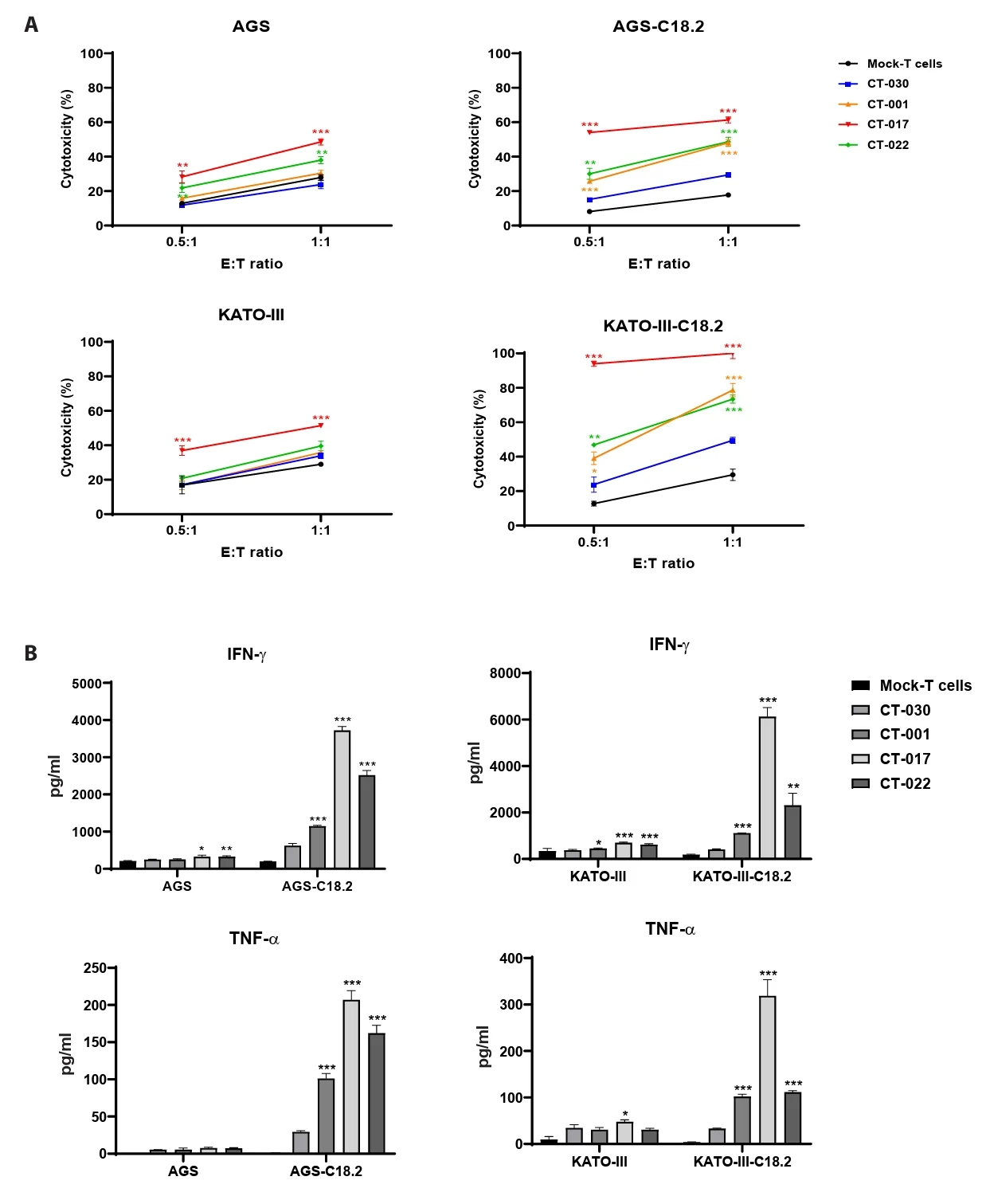
Cytotoxicity and cytokine release of CLDN18.2 CAR-T cells in the presence of gastric cancer cells. (A) Cytotoxicity of CLDN18.2 CAR-T cells. CLDN18.2 CAR-T or Mock-T cells were co-cultured with gastric cancer cells at effector-to-target (E:T) ratios of 0.5:1 and 1:1 for 24 h. In all panels, Mock‑T cells indicate non‑transduced, bead‑activated T cells that were not infected with lentiviral vectors and therefore serve as a reference for antigen‑independent T‑cell activity. LDH was detected in the supernatants of CAR-T cells co-cultured with target cells. Data are expressed as the Mean ± SD (n = 3), and p-values were calculated using post-hoc tests after ANOVA. (B) Cytokine release capacity of CLDN18.2 CAR-T cells. CLDN18.2 CAR-T or Mock-T cells were co-cultured with target cells at an E:T ratio of 1:3 for 24 h. The concentrations of IFN-γ and TNF-α in the supernatant of CAR-T cells co-cultured with target cells were determined by Elisa. Data are expressed as the Mean ± SD (n = 3), and p-values were calculated using post-hoc tests after ANOVA. ^*^p < 0.05, ^**^p < 0.01, ^***^p < 0.001.

To assess the antitumor efficacy of CLDN18.2 CAR-T cells in vitro, cytotoxic activity was quantified by measuring LDH release after co-culturing CAR-T or Mock-T cells with gastric cancer lines (AGS, AGS-C18.2. KATO III, and KATO III-C18.2). As shown in Fig. 3A, all CLDN18.2 CAR-T variants (CT-030, CT-001, CT-017, and CT-022) displayed significantly enhanced CLDN18.2 antigen-specific cytotoxicity compared to Mock-T cells. Constructs containing the PD-1/CD28 CSR and/or CypA-specific CT-001, 017, and CT-022 exhibited superior cytotoxic potency compared to the base CT-030 CAR-T cells.

Cytokine secretion profiles were concurrently evaluated for IFN-γ and TNF-α production upon co-culture with the same CLDN18.2-expressing gastric cancer cell lines. All engineered CLDN18.2 CAR-T cells showed robust increases in IFN-γ and TNF-α release compared to Mock-T controls. The highest induction was observed in CT-001, CT-017, and CT-022 cells, validating their incorporation of PD-1/CD28 CSR and/or CypA (Fig. 3B).

Collectively, these findings demonstrate that the CLDN18.2 CAR constructs PD-1/CD28 CSR and CypA improve CLDN18.2-specific cytotoxicity and pro-inflammatory cytokine secretion in vitro, consequently facilitating enhanced antitumor activity against gastric cancer cells.

### Antitumor efficacy of CLDN18.2 CAR-T cells in a gastric cancer mouse model

**Fig. 4.**
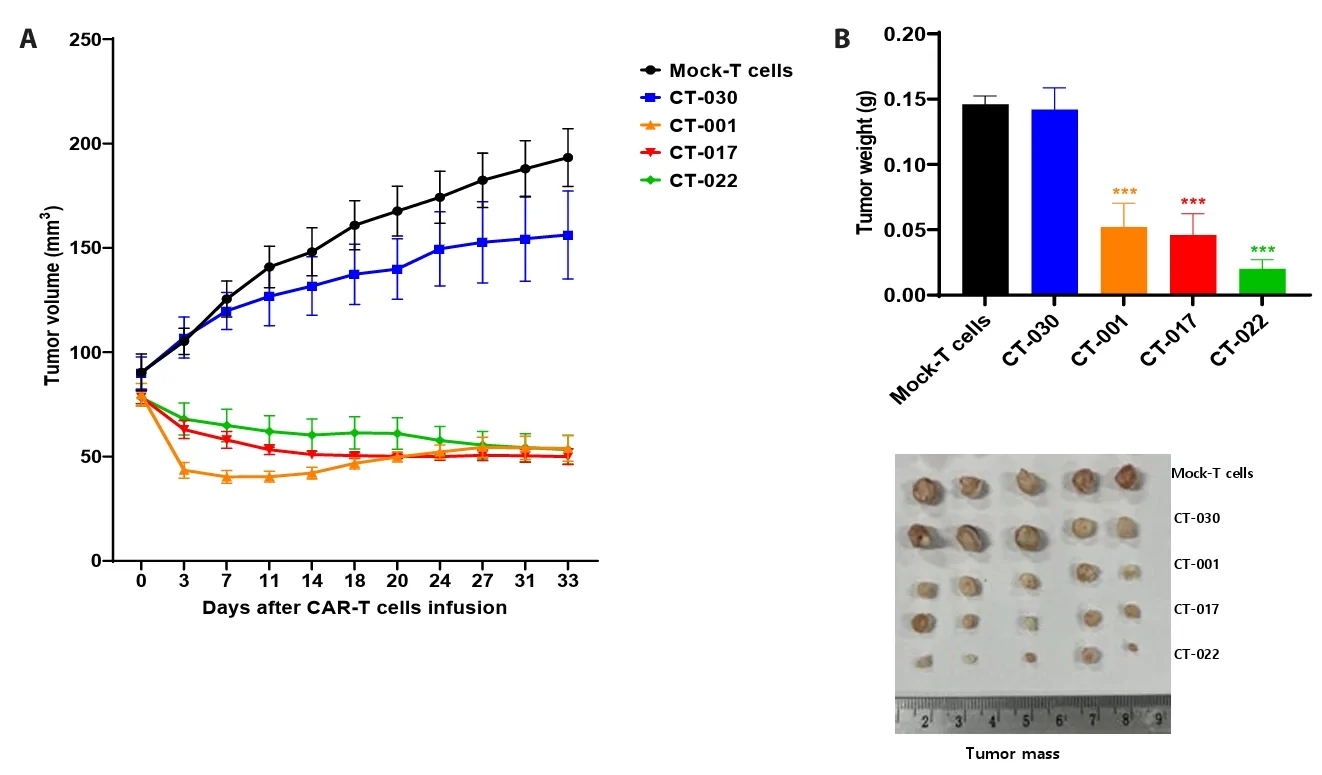
The efficacy of CLDN18.2 CAR-T cells in a mouse model of gastric cancer. After establishing the gastric cancer mouse model, CAR-T cells or Mock T cells were intravenously injected into the mice. Mock-T cells were non-transduced controls, allowing differentiation between background and specific CAR-dependent anticancer efficacy. (A) Tumor volume. The anticancer efficacy of CAR-T cells in vivo was evaluated by measuring the tumor volume and body weight of the mice twice per week. Tumor volumes decreased over time in all mice in the CLDN18.2 CAR-T cell-administered group. In particular, CT-001, 017, and 022 CAR-T cells significantly diminished tumor volume compared to the CT-030 and Mock-T cell groups. (B) Tumor weight. After euthanasia, tumor masses were isolated and weighed. Tumor weights markedly decreased in CT-001-, 017, and 022 CAR-T-administered mice; 022 CAR-T cells further reduced tumor weight compared to the other cells. CypA only CLDN18.2 CAR T cells were not included, as the construct was not engineered in the present study.

**Fig. 5.**
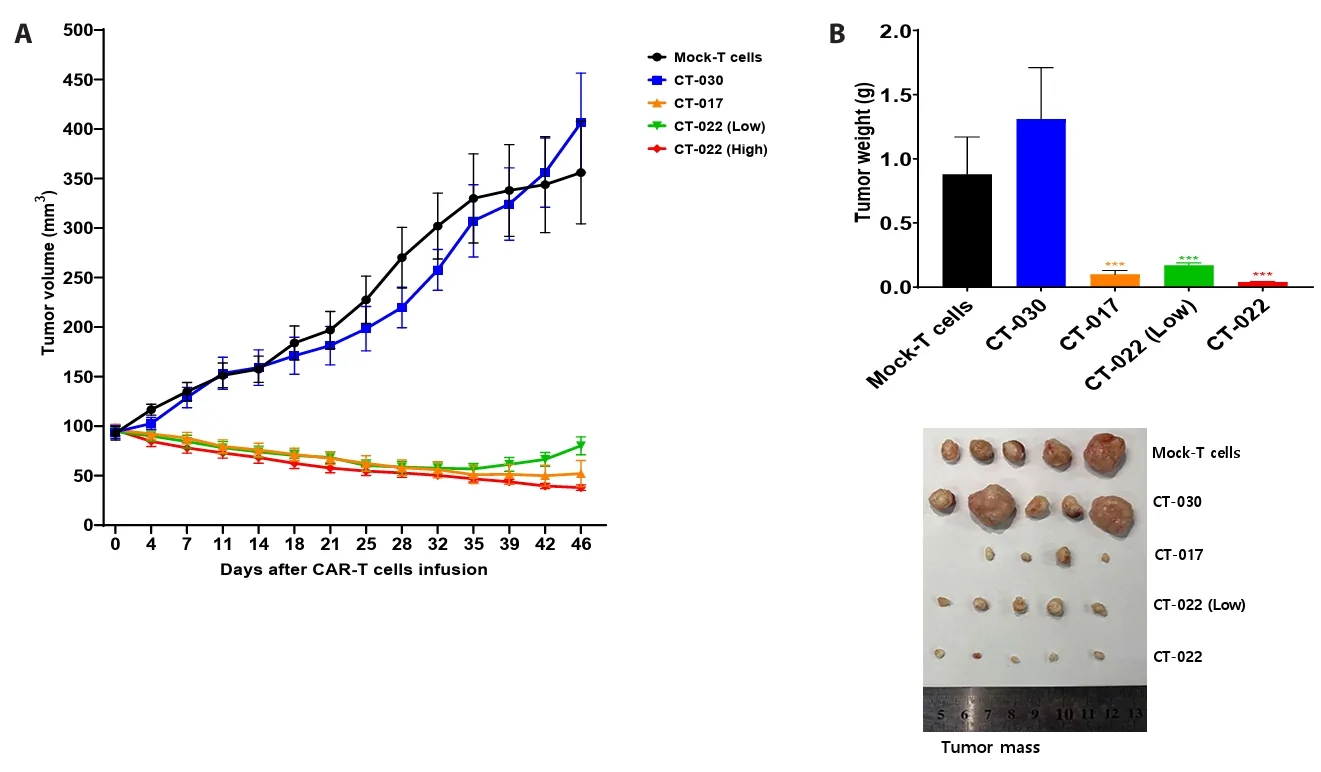
The effectiveness of CT-017 and CT-022 CAR-T cells in a mouse model of gastric cancer. (A) Tumor volume. To evaluate the effectiveness of CT-017 and CT-022 CAR-T cells in vivo, a low dosage of CT-022 cells (0.5 × 10^6^ cells) as well as 5 × 10^6^ CT-022, CT-017, and Mock-T cells were inoculated into the gastric cancer mouse model. Anticancer effects were determined by measuring changes in tumor volume and body weight at 46 days after CAR-T cell administration. Tumor volume and weight in mice within the CT-017 and 022 CAR-T cell-inoculated groups significantly decreased compared to those in the Mock-T cell- and CT-030-inoculated groups. The ability of CT-017 and 022 CAR-T cells to decrease tumor volume was maintained until the endpoint of the experiment (on day 46 post-injection). In particular, CT-022 resulted in a more effective and lasting decrease in tumor volume than that in other cells. (B) Tumor weight. The mice were euthanized on day 46 after CAR-T cell inoculation, and the tumor masses were weighed. Tumor weights were markedly reduced in CT-017 and CT-022 CAR-T cell-administered mice; 022 CAR-T cells further reduced tumor weight compared to the other cells.

To evaluate antitumor efficacy in vivo, gastric cancer xenograft models were established through subcutaneous injection of AGS-C18.2 cells into mice, followed by intravenous administration of either CLDN18.2 CAR-T or Mock-T cells. Tumor volume and body weight were serially monitored as primary endpoints. All CLDN18.2 CAR-T–cell groups exhibited a progressive reduction in tumor volume compared to the controls (Figs. 4 and 5). CAR-T variants incorporating the PD-1/CD28 CSR—CT-001, CT-017, and CT-022—demonstrated substantially enhanced antitumor activity compared to the base construct CT-030. CT-022, which additionally carried CypA, achieved the greatest suppression of tumor growth across both the short- and long-term observation periods.

No significant changes in body weight or unexpected mortality were observed in the CAR-T or Mock-T cell groups. This indicates that the systemic administration of CAR-T cells did not cause overt toxicity in vivo (data not shown). Mice were euthanized, and tumor masses were isolated and weighed at the study endpoints (day 33, Fig. 4; day 46, Fig. 5). Tumor weights were significantly reduced in all groups receiving CT-001, CT-017, or CT-022 CAR-T cells compared to those in the Mock-T and CT-030 groups. CT-022 exhibited the most pronounced decrease of approximately 88% (Fig. 4) and 98% (Fig. 5), respectively, compared to CT-030.

These data demonstrate that CLDN18.2 CAR-T cells equipped with the PD-1/CD28 CSR exert superior antitumor efficacy, and that the additional incorporation of CypA into CT-022 further potentiates tumor suppression in a gastric cancer xenograft model. A sustained reduction in tumor volume and mass in the absence of treatment-related toxicity validates the therapeutic potential of the CT-022 design for advanced gastric cancer.

## Discussion

Despite major advances in recent years, the treatment of solid tumors with CAR-T cell therapy remains challenging. More than 50% of patients relapse after CAR-T cell infusion, underscoring key barriers such as immunosuppressive tumor microenvironments, infiltration defects, and T-cell exhaustion. These limitations have encouraged the development of next-generation CAR-T constructs capable of enhanced signaling and immune modulation. CypA has emerged as a pivotal contributor in T-cell receptor signaling and cytokine regulation, with multiple studies showing its potential to improve antitumor immune responses (Anto et al., 2022; Dawar et al., 2017; Kalinina et al., 2021). Therefore, we adopted a modular engineering strategy that enabled the introduction of CypA and switch receptors into CAR-T vectors for flexible, multitarget attacks against immune-resistant tumor niches.

Interestingly, the in vivo antitumor activity of CT‑022 did not fully mirror the in vivo ranking observed in Fig. 3, where CT‑017 showed slightly higher LDH‑based cytotoxicity and cytokine release than CT‑022. These short‑term assays mainly capture an early effector burst driven by PD‑1/CD28 signaling and therefore may underestimate the advantages of the tri‑cistronic configuration. In CT‑022, the addition of CypA to the ICOS‑based CAR and PD‑1/CD28 CSR backbone is designed to amplify ZAP70–calcineurin–NFAT signaling, sustain IL‑2, IFN‑γ, and TNF‑α transcription, and enhance T‑cell proliferation and survival under repeated antigen stimulation. This long‑term signaling reinforcement likely explains why CT‑022 ultimately exerts superior tumor control in vivo compared with CT‑017, despite its slightly lower peak activity in 24‑h in vitro readouts.

CLDN18.2 targeting is a particularly promising approach in advanced gastric and pancreatic cancers, wherein poor response to conventional therapy and late detection result in high mortality. Epidemiological data highlight this clinical urgency. Gastric cancer is among the most lethal cancers worldwide, with a 5-year survival rate of approximately 30% for metastatic or recurrent cases; similar challenges are observed in pancreatic cancer. CLDN18.2, a tight junction protein that is almost exclusively expressed in malignant gastrointestinal tissue, has shown consistent overexpression in 60–90% of tumor samples across multiple clinical trials. Our multifunctional platform exploits this specificity by targeting CLDN18.2 in the context of real-world clinical diversity and molecular complexity.

**Fig. 6.**
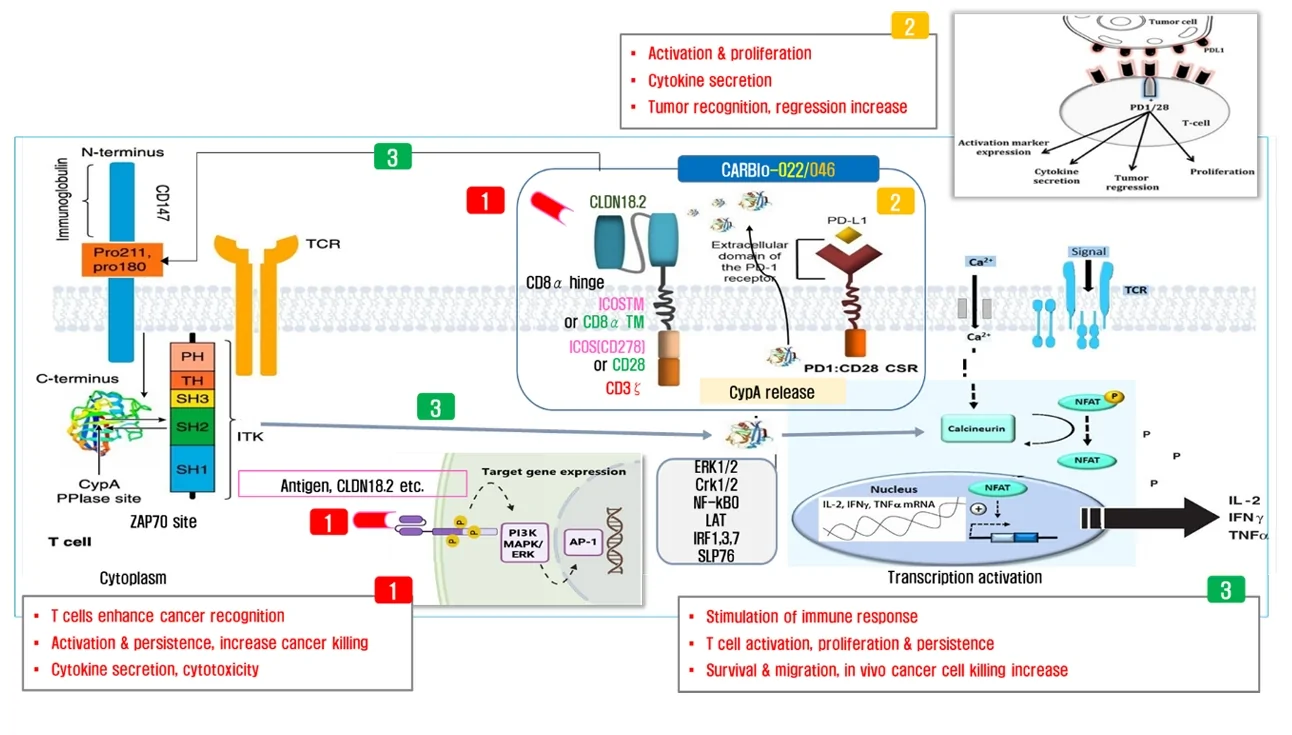
CARBio Next-Generation CAR-T Platform Technology and Cell Function. Tri-cistronic CAR-T cells kill cancer cells by continuously activating CAR-T cells with minimal side effects. First, reprogrammed CAR-T cells can target gastric cancer cells more precisely by binding to the CLDN18.2 antigen receptor. Second, modular signaling induces strong T-cell activation and immune responses. Switch molecules are expressed along with CAR-T signaling to control immune cell suppression mechanisms and enhance CAR-T cell activation and anticancer efficacy. Third, CypA secretion promotes T-cell activation through SH2 phosphorylation at the ZAP-70 site, which is required for signaling to the T-cell receptor terminal. After translocation to the cytoplasm, it enables NFAT dephosphorylation of calcineurin and facilitates its translocation into the nucleus. The transcriptional initiation of NFAT in the nucleus activates IL-2 cytokine secretion, prolongs T cell survival, helps maintain function in the body, and activates T cells.

Mechanistically, our tri-cistronic CAR-T cell system incorporates ICOS as the co-stimulatory domain. This domain stabilizes T-cell persistence and reduces the risk of cytokine release syndrome. In contrast, PD-1/CD28 CSR and CypA enhance T-cell activation, signaling adaptation, and resistance to exhaustion. The functional mechanism of our tri-cistronic CAR-T platform is illustrated in Fig. 6, which visually integrates the molecular architecture and cell signaling pathways that drive the enhanced antitumor performance. The schematic highlights three core modules: a CLDN18.2-targeting extracellular domain for specific tumor recognition, an ICOS (or CD28)-based co-stimulatory domain, a PD-1/CD28 switch receptor to mitigate immune checkpoint-mediated suppression, and the inclusion of CypA to amplify intracellular signaling and persistence. Upon antigen engagement, coordinated activation of TCR and CAR elements leads to downstream events, including ZAP70 phosphorylation, calcineurin-mediated NFAT translocation, and robust transcriptional activation of cytokines such as IL-2, IFN-γ, and TNF-α. The modular design not only stimulates cytotoxicity and proliferation but also ensures sustained immune responses and effective tumor elimination in vivo. Figure 6 illustrates the mechanisms through which the multilayered integration of signal regulators and molecular switches within the CARBio platform mitigates conventional barriers to CAR-T efficacy in solid tumor models. Our results demonstrate that the tri-cistronic configuration provides superior tumor control, as evidenced by sustained cytotoxicity both in vivo and in vivo, increased IFN-γ and TNF-α secretion, and substantial tumor regression in murine models compared to those in conventional CAR-T platforms (Figs. 3–5). Importantly, the incorporated molecular switches function only upon CAR engagement, thereby reducing off-target toxicity and maximizing safety, which is a key challenge in this field (Blaeschke et al., 2021; Lee et al., 2024).

The discrepancy between the in vivo and in vivo profiles of CT‑017 and CT‑022 is also consistent with the distinct demands imposed by the tumor microenvironment. In xenograft models, CAR‑T cells experience chronic PD‑L1 engagement, nutrient deprivation, hypoxia, and immunosuppressive cytokines, conditions under which CypA‑mediated maintenance of NFAT activity and IL‑2 production provides a survival and persistence advantage. CT‑017 appears to deliver a strong initial effector burst but lacks this additional layer of support, which may accelerate proliferative arrest and exhaustion over time. In contrast, CT‑022 maintains functional CAR‑T cell numbers and effector function for longer periods, resulting in more profound and durable tumor regression, including at lower cell doses. Taken together, these observations indicate that CT‑017 and CT‑022 differ primarily in long‑term fitness rather than in per‑cell killing potency. Single‑time‑point LDH and cytokine assays favor constructs with strong early effector responses, whereas long‑term tumor models are more sensitive to traits such as proliferative capacity, resistance to exhaustion, and adaptation to an immunosuppressive tumor microenvironment. Our data therefore suggest that the tri‑cistronic design of CT‑022 confers a clear advantage in durability and low‑dose efficacy, even if its short‑term in vivo cytotoxicity does not always exceed that of CT‑017.

A methodological limitation of this study is the use of non-transduced, bead-activated T cells as Mock-T controls, which, while sufficient to define CLDN18.2-independent background activity, does not account for vector-related effects such as lentiviral transduction stress or clonal selection. Moreover, we did not include a CLDN18.2 CAR-T variant expressing CypA in the absence of the PD-1/CD28 switch, preventing isolation of CypA’s individual contribution. Future work will incorporate mock-transduced controls with empty or antigen-negative vectors and construct PD-1/CD28-negative, CypA-positive CAR-T cells to disentangle the respective and synergistic effects of these modules on antitumor efficacy within the tri-cistronic platform.

Collectively, these innovations enable our platform to target immunosuppressive pathways without additional drug administration, thereby achieving robust and durable antitumor effects. The tri-cistronic CAR-T design represents a major conceptual advance. It provides a versatile, modular scaffold that not only enhances targeting and effector function but also establishes a basis for next-generation cellular immunotherapies against solid tumors. With promising preclinical and early clinical evidence for CLDN18.2-targeted therapies, this approach will have a significant translational impact on the landscape of immunotherapy-resistant cancers.

## Supplementary Information

Fig. S1. The PD‑L1 expression on the gastric cancer cell lines. PD-L1 expression levels on AGS, AGS-CLDN18.2, KATO III, and KATO III-CLDN18.2 cells were measured by flow cytometric analysis after staining with PE/Cyanine7 anti-human PD-L1 antibody (BioLegend). The expression of PD-L1 was detected in approximately 45% of AGS cells, 10% of AGS-CLDN18.2 cells, 45% of KATO III cells, and 43% of KATO III-CLDN18.2 cells. These results suggest that the gastric cancer cells used in this study provide endogenous PD‑L1 ligands for PD‑1/CD28 engagement, albeit at moderate levels.

Fig. S2. Proteotyping analysis based on the amino acid sequence of the HVR in the Cap gene. The maximum likelihood phylogenetic tree for each protein (Left) is shown. The color assigned to each amino acid is indicated top. The blank and consensus sequences are represented in black and white, respectively. The proteotypes of HVR in the Cap gene (Right) are represented by numbers, and each proteotype is clearly distinguished by a specific color.
