# Supplementary material for: Development of tri-cistronic CLDN18.2 CAR-T cells incorporating PD-1/CD28 switch and cyclophilin A for enhanced solid tumor immunotherapy
Source: J Microbiol. 2026 Jan 31;64(1):e2510017. doi: 10.71150/jm.2510017 (PMC13577791; doi:10.71150/jm.2510017)
Supplement: Supplementary file 1 [file jm-2510017-Supplementary-Fig-S1.pdf]

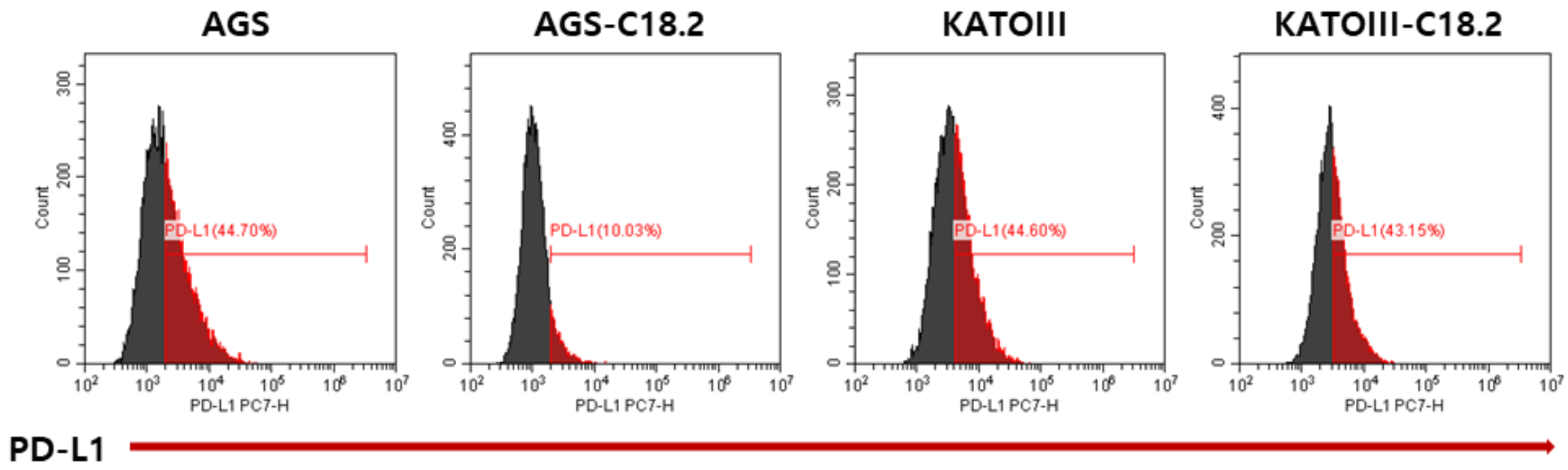

Fig. S1. The PD-L1 expression on the gastric cancer cell lines. PD-L1 expression levels on AGS, AGS-CLDN18.2, KATO III, and KATO III-CLDN18.2 cells were measured by flow cytometric analysis after staining with PE/Cyanine7 anti-human PD-L1 antibody (BioLegend). The expression of PD-L1 was detected in approximately 45% of AGS cells, 10% of AGS-CLDN18.2 cells, 45% of KATO III cells, and 43% of KATO III-CLDN18.2 cells. These results suggest that the gastric cancer cells used in this study provide endogenous PD-L1 ligands for PD-1/CD28 engagement, albeit at moderate levels.
