# Supplementary material for: Development of tri-cistronic CLDN18.2 CAR-T cells incorporating PD-1/CD28 switch and cyclophilin A for enhanced solid tumor immunotherapy
Source: J Microbiol. 2026 Jan 31;64(1):e2510017. doi: 10.71150/jm.2510017 (PMC13577791; doi:10.71150/jm.2510017)
Supplement: Supplementary file 2 [file jm-2510017-Supplementary-Fig-S2.pdf]

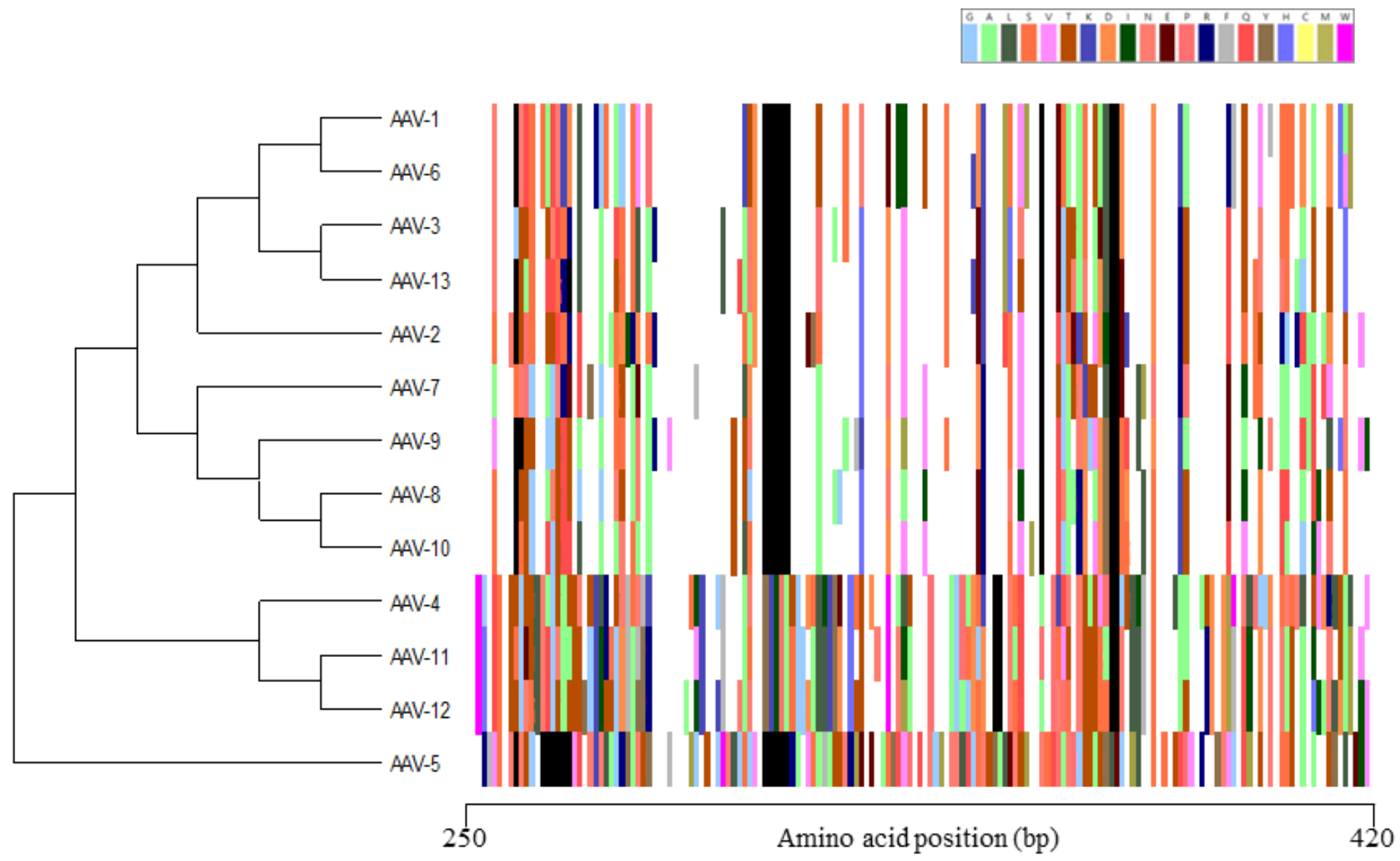

Types	HVR
AAV-1	1
AAV-6	1
AAV-3	2
AAV-13	3
AAV-2	4
AAV-7	5
AAV-9	6
AAV-8	7
AAV-10	8
AAV-4	9
AAV-11	10
AAV-12	11
AAV-5	12
Total proteotypes	12

Fig. S2. Proteotyping analysis based on the amino acid sequence of the HVR in the Cap gene. The maximum likelihood phylogenetic tree for each protein (Left) is shown. The color assigned to each amino acid is indicated top. The blank and consensus sequences are represented in black and white, respectively. The proteotypes of HVR in the Cap gene (Right) are represented by numbers, and each proteotype is clearly distinguished by a specific color.
